# Does Sex or Age Impact the Prognostic Value of a Zero Coronary Artery Calcium Score?

**DOI:** 10.3390/jcm14176260

**Published:** 2025-09-04

**Authors:** Jeffrey L. Anderson, Dave S. Collingridge, Viet T. Le, Leslie Iverson, Joseph B. Muhlestein, Tami L. Bair, Stacey Knight, Steve M. Mason, Kirk U. Knowlton

**Affiliations:** 1 Intermountain Heart Institute, Intermountain Health, Salt Lake City, UT 84107, USA; dave.collingridge@imail.org (D.S.C.); viet.le@imail.org (V.T.L.); leslie.iverson@imail.org (L.I.); brent.muhlestein@imail.org (J.B.M.); tami.bair@imail.org (T.L.B.); stacey.knight@imail.org (S.K.); steve.mason@imail.org (S.M.M.); kirk.knowlton@imail.org (K.U.K.); 2 Department of Medicine, University of Utah, Salt Lake City, UT 84112, USA; 3 Rocky Mountain University of Health Professionals, Provo, UT 84606, USA

**Keywords:** age, coronary artery calcium, coronary artery disease, prognosis, risk factors, women

## Abstract

**Background:** It is unclear whether sex or age impacts the prognostic value of a zero coronary artery calcium (CAC) score. **Methods**: We searched our electronic medical record (eMR) database for primary prevention patients who underwent positron emission tomography/computed tomography (PET/CT) stress testing. We assessed coronary prognosis and all-cause death during 2.2 (SD 1.9) years of follow-up in women vs. men and in those ≥65 vs. <65 years old by CAC = 0 vs. CAC > 0 scores. **Results**: We identified 40,018 qualifying patients, of which 48.7% were women and 58.9% were ≥65. CAC = 0 was present in 7967 (19.9%), of which 67.8% were women, and 34.9% were aged ≥65. In CAC = 0 patients, 13 coronary events occurred: 7 (0.13%) in women and 6 (0.24%) in men (*p* = 0.28); and 6 (0.12%) in <65 and 7 (0.25%) in ≥65 years old (*p* = 0.15). All-cause death rates comparing CAC = 0 to CAC > 0 subjects were 3.1% vs. 9.8% overall: 3.1% vs. 9.5% in women and 3.3% vs. 10.2% in men, 2.4% vs. 6.9% for ages <65, and 4.7% vs. 11.5% for ≥65 years old; all *p* < 0.001. **Conclusions**: A zero CAC score predicts an excellent prognosis for not only coronary events but also all-cause mortality, both overall and in women and the elderly.

## 1. Introduction

There is general agreement that a coronary artery calcium (CAC) score of zero predicts a very low 5-year risk of coronary events [1,2,3,4]. However, there is limited information on whether the prognostic value of CAC = 0 varies by sex [5,6]. For example, non-atherosclerotic causes of coronary ischemia present more often in women, including spontaneous coronary artery dissection [7] and microvascular angina [8,9,10], which are generally not predicted by coronary artery calcium (CAC), and may contribute an uncertain proportion to coronary events. Further, CAC imaging is often performed at an older age in women. Whether these factors impact the prognostic utility of CAC = 0 in women vs. men is uncertain.

Further, information as to whether CAC = 0 remains a strong predictor of prognosis in older individuals also is limited [5,6]. Age contributes strongly to the prediction of cardiovascular risk in risk-factor-based models such as the pooled cohort and PREVENT equations [11,12], with risk increasing markedly at older ages. The age of subjects in most reports on clinical applications of CAC has averaged in the 40s or 50s [13]. Whether the usefulness of a zero CAC score diminishes as individuals age, because of a variety of age-related factors independent of CAC, is unclear, including the usefulness of risk-based equations to guide the selection of statin therapy. Our goal in this study was to address these uncertainties based on a large CAC cohort in an integrated healthcare system.

## 2. Materials and Methods

Study Design and Objectives and IRB Approval: This was a retrospective, observational study. The study objectives were (1) to define the prevalence of CAC = 0 in patients at risk of primary coronary artery disease (CAD) referred for a stress PET/CT study for symptoms potentially cardiovascular (CV) in nature, or for the need for definitive, pre-intervention risk assessment (e.g., before non-coronary CV surgery, renal transplantation, or antiarrhythmic drug initiation); (2) in CAC = 0 subjects, to determine absolute coronary event rates (defining ≈0.1%/year as very-low risk), and then to compare these outcomes of CAC = 0 subjects by sex and by age over an intermediate follow-up time; (3) to compare all-cause mortality over 2.2 years, comparing CAC = 0 vs. CAC > 0 overall, by sex and by age ≥65 vs. <65 years, in both unadjusted and risk-factor adjusted analyses.

This retrospective database study was conducted in accordance with the Declaration of Helsinki. This study was approved with a waiver of consent by the Institutional Review Board of Intermountain Health (protocol code 1007205, approval date 23 August 2023).

Study Populations: To identify the study population, the Intermountain Health’s nuclear medicine and hospital electronic medical records (eMR) databases at 4 hospitals were searched for patients undergoing stress PET/CT between February 2016 and July 2022 and who were at primary atherosclerotic cardiovascular disease risk (i.e., patients with no prior history of MI, stroke, or coronary intervention). Of these, the subset with CAC = 0 was identified.

We divided the database of CAC = 0 patients by sex (women, men) and age (<65, ≥65 years) at PET/CT testing. We compared demographics and outcomes between these patient subsets. The primary outcomes were coronary death, non-fatal MI, or all-cause death.

PET/CT and CAC Protocols: The technical features of our stress PET/CT studies have been previously published [14,15]. Here, we focus on the CT results, which yielded CAC scores. Following initial rest perfusion imaging, CAC was assessed using a non-enhanced 16-slice CT scanner triggered by prospective electrocardiogram gating, which generated 3 mm thick image slices during breath-holding. CAC scores were calculated by the Agatston method [16]. Stress perfusion imaging then was performed using regadenoson as the pharmacologic stressor. CAC results were taken from clinical readings of nuclear and CT board-certified or qualified cardiologists who were unaware of the study aims or results.

Study Outcomes: Consistent with the study objectives, we assessed the percentage of total CT studies with CAC = 0 and CAC > 0. We then verified coronary events (i.e., coronary death or non-fatal MI) during follow-up in CAC = 0 patients by chart review. We compared the outcomes overall and then by sex and by age <65 versus ≥65 years.

Because specific causes of death were not available in CAC > 0 patients, we also compared all-cause deaths by CAC category, both overall and by sex and age subsets in both unadjusted and multivariate-adjusted analyses.

Statistical Considerations: Variables of interest are summarized as means +/− SD or as medians and interquartile ranges as appropriate. We compared demographic and other baseline features of CAC = 0 subjects by sex and by age categories. Categorical variables were compared by chi-square analysis and continuous variables by *t*-test. The relationship between CAC status (0 vs. >0) and all-cause death was assessed with binary logistic regression while adjusting for 9 covariates, including age, sex, and 7 key cardiovascular and non-cardiovascular risk factors. A 2-sided *p*-value of ≤0.05 was taken to be significant.

## 3. Results

### 3.1. Study Population and Baseline Characteristics

Our database search identified 40,018 patients who were at primary coronary risk and who had undergone stress PET/CT studies. Of these, 7967 (19.9%) had a zero CAC score on CT. Overall, 19,495 were women (48.7%) and 20,523 (51.3%) were men; age averaged 65.8 years (SD 11.9), with 41.1% <65 and 58.9% ≥65 years old. Of CAC = 0 subjects, 5400 (67.8%) were women and 2567 (32.2%) were men. Age in CAC = 0 patients averaged 60.5 years (SD 12.0) in women and 53.8 y (SD 12.6) in men. Dividing the CAC = 0 population by age resulted in 5185 who were <65 (65.1%) and 2782 (34.9%) who were ≥65 years old.

Table 1 shows the baseline characteristics of CAC = 0 patients by sex. Women were older and more frequently had a family history of heart disease and a history of hyperlipidemia, stroke, and depression. Men more frequently had a history of smoking and heart failure.

Baseline characteristics of CAC = 0 patients by age are shown in Table 2. Median ages were 52.0 and 70.0 years in the two subsets, respectively. Subjects in the older set more frequently were women and more often had a history of hyperlipidemia, hypertension, atrial fibrillation, COPD, and heart failure, but they less often were smokers or had a diabetes diagnosis.

### 3.2. Clinical Outcomes

**Figure 1:**
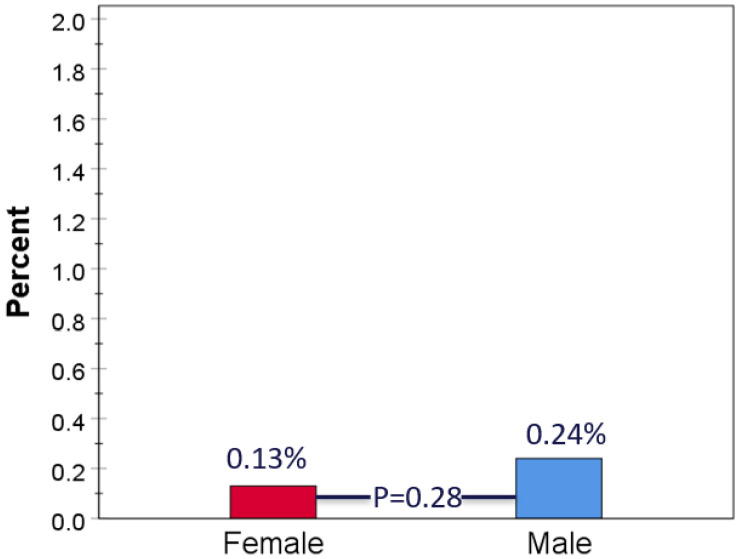
MI and coronary deaths in CAC = 0 patients by sex in CAC = 0 cohort. Events per cohort population for females and males during follow-up were 7/5400 and 6/2782, respectively.

**Figure 2:**
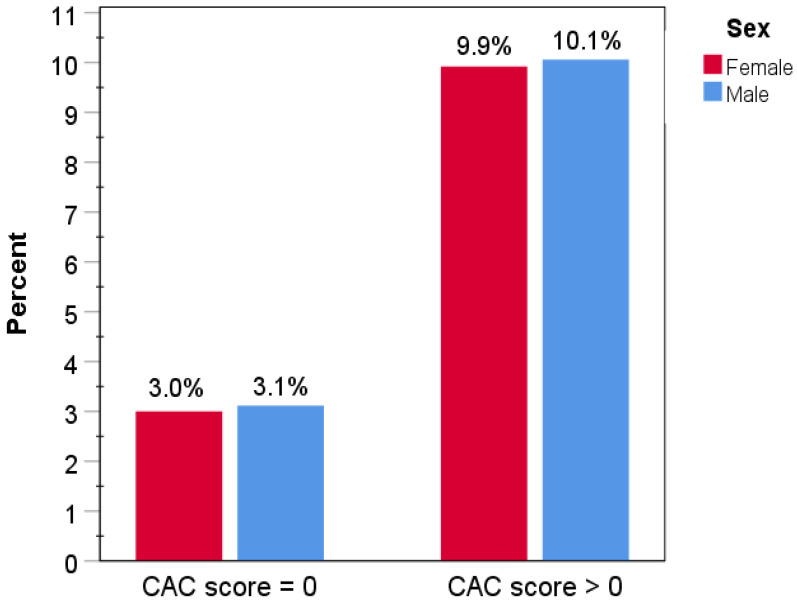
All-cause death by CAC score and sex. Percentages of all-cause death by CAC category and sex during follow-up.

**Figure 3:**
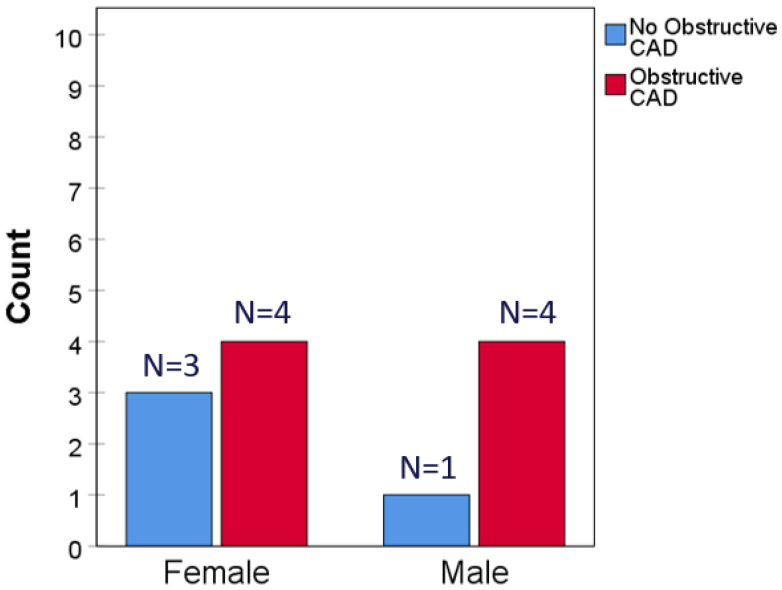
MI with or without obstructive CAD by sex in CAC = 0 cohort. Number and proportions of MI events associated with or without obstructive CAD are given for female and male participants, respectively, *p* = 0.31.

During a follow-up of 2.22 (SD 1.85) years, a total of 13 primary events occurred, including 12 non-fatal MI’s and 1 coronary death. By sex, seven events (0.13%) occurred in CAC = 0 women and six (0.24%) in CAC = 0 men (*p* = 0.28) (Figure 1). Rates of all-cause death comparing CAC = 0 to CAC > 0 subjects were 3.0% vs. 10.0% overall; by sex they were 3.0% vs. 9.9% in women and 3.1% vs. 10.1% in men (all *p* < 0.001; Figure 2). CAC remained a highly significant independent predictor of all-cause mortality overall and in both sexes after adjusting for age and seven cardiovascular or non-cardiovascular risk factors (*p* < 0.001; Supplementary Table S1). MI with no obstructive CAD (MINOCA) constituted 3/7 MIs in women and 1/5 MIs in men (*p* = 0.31; Figure 3).

**Figure 4:**
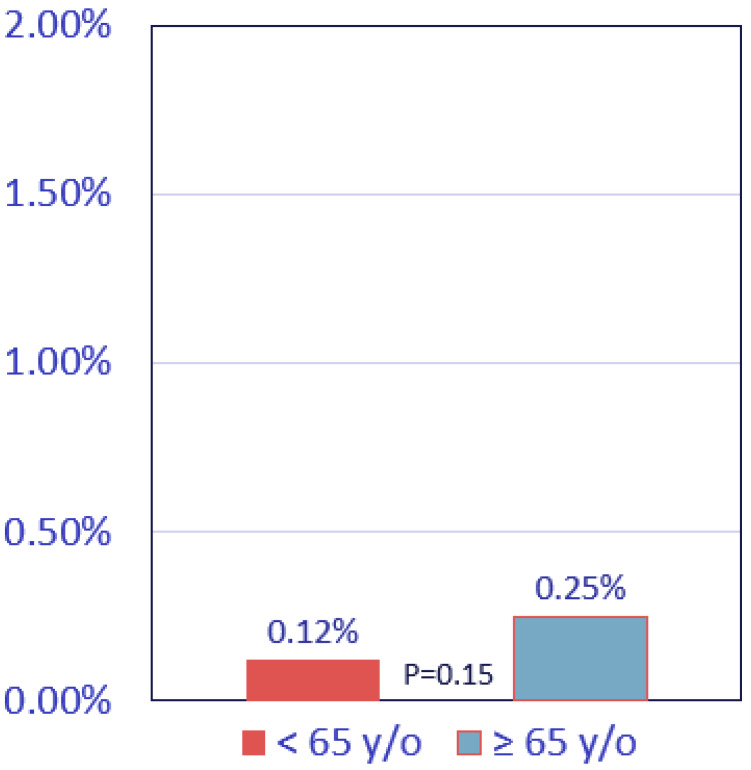
MI or coronary death by age in CAC = 0 patients. Rates of non-fatal MI or coronary death during follow-up are given by age category in CAC = 0 cohorts.

**Figure 5:**
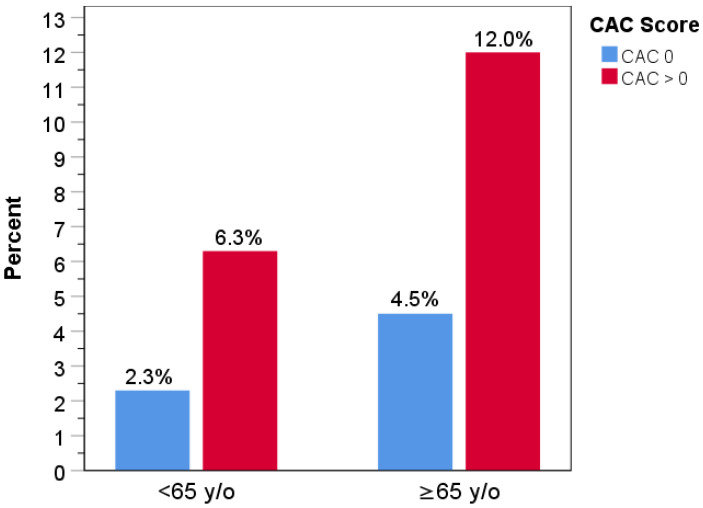
Rates of all-cause death by age and CAC score. Percentages of all-cause death by CAC category and age are shown during follow-up.

Comparing events by age <65 vs. ≥65 yielded 6 events (0.12%) vs. 7 (0.25%) events, respectively (*p* = 0.15) (Figure 4). All-cause death rates by age, comparing CAC = 0 with CAC > 0 subjects, were 2.3% vs. 6.3% for age <65 and 4.5% vs. 12.0% for age ≥65 years old (both *p* < 0.001, Figure 5). CAC remained a highly significant independent predictor of all-cause mortality overall and in both age groups after adjusting for sex and seven other major cardiovascular and non-cardiovascular risk factors (*p* < 0.001 for all comparisons; Supplementary Table S1). CAC > 0 emerged as the strongest independent predictor of death of these risk factors overall, with an adjusted odds ratio of 2.31 (95% CI 2.00–2.66). Similarly, it was independently predictive of mortality for all sex and age subsets with an adjusted OR range of 2.18–2.35 (Supplementary Table S1).

## 4. Discussion

Summary of Study Findings. Women and the elderly generally are underrepresented in clinical trials and observational studies. To what extent a CAC = 0 predicts a low risk of coronary events and CV and all-cause mortality in women and in the elderly deserves additional assessment. In this large, integrated healthcare system cohort with CT assessments of CAC, we found that a CAC = 0 is frequently found in these underrepresented patient subsets and predicts an excellent coronary prognosis, regardless of sex or age. Further, this excellent prognosis extends to all-cause mortality. Indeed, CAC > 0 was associated with a 3-fold increase in mortality risk, and this risk persisted after extensive multivariable adjustment. Indeed, CAC > 0 represented the strongest independent predictor of death (adjusted OR = 2.3). These findings suggest more extended use of CAC testing overall, in women as well as in men, and in older as well as in younger patients, in whom more precise risk assessment is indicated for both coronary events as well as for overall mortality.

Comments on Study Objectives and Comparisons. The primary objective of this study was to determine absolute coronary event rates (defining ≈0.1%/year as very-low risk [1]) and then to compare these outcomes between subgroups of CAC = 0 patients by sex and by age rather than to compare them to CAC > 0 cohorts. There already are abundant studies showing that CAC = 0 carries a much better prognosis for coronary events than CAC > 0 [1,2,3,4], and coronary event and cause-specific death determination were not available to us in the very large CAC > 0 population. However, all-cause death information was available in both CAC subsets, and we performed not only unadjusted comparisons but also multivariate logistic regression comparisons between CAC = 0 and CAC > 0 subsets, adjusting for nine common coronary and all-cause mortality risk factors (Supplementary Table S1). As we report, CAC > 0 emerged as a very strong and independently contributing risk factor for all-cause death. Thus, CAC = 0 not only predicts a very low coronary risk, independent of sex and age, but also is a marker of general vascular and non-vascular health and improved survival.

Literature Comparisons and Mechanistic Considerations. CAC has been recognized as a risk factor for 3 decades [17], yet its general clinical application remains limited. It is currently used as a secondary risk factor, i.e., as a “tie-breaker” or “risk-enhancing factor” to use in those at intermediate or indeterminate risk by risk-factor equation assessment [18]. However, literature reports specifically addressing the utility of CAC in women and by age are limited [5,6].

Frey et al. assessed the predictive power of zero CAC at PET/CT study in 2640 patients, 26% with zero CAC [5]. Age averaged 65 years, and 46% were women. Zero CAC was generally predictive of a normal stress PET scan, irrespective of age and sex.

The MESA (Multiethnic Study of Atherosclerosis) investigators enrolled 6722 men and women, representing four racial and ethnic groups and assessed the predictive ability of CAC for coronary events [1]. CAC score strongly predicted incident coronary heart disease events and provided incremental information beyond the standard risk factors, both overall and among each of the included racial/ethnic groups.

Blaha et al. subsequently assessed the change in risk caused by the addition of each of 13 new negative risk markers using diagnostic likelihood ratios [6]. A CAC score of zero emerged as showing the strongest negative shift in estimated CV disease risk. CAC = 0 was useful in the 3601 women as well as in men, and it was also of value in older individuals.

We tested the utility of CAC as a primary risk predictor in selecting 600 subjects for statin therapy in a randomized pilot study [19]. Statins were recommended less often, but compliance and lipid-lowering were better in the CAC arm than the pooled cohort equation arm.

Mechanistically, CAC is an anatomic marker of disease burden, which by itself integrates both known and unknown risk factors (factors which are probabilistic in nature with variable penetrance at the level of individual patients). We and others therefore use a CAC = 0 score as an indicator of very low coronary risk, largely independent of probabilistic risk factors, at least over a span of about 5 years [1,19].

It has been argued that the presence of “soft plaque”, not detected by CAC scan, could limit the prognostic utility of CAC = 0 [20,21]. Mittal et al. assessed the prevalence of obstructive CAD and prognosis in patients with stable symptoms and a zero CAC score [22]. They reported that obstructive CAD was rare, and prognosis was excellent, including when non-calcified atheroma was identified. They argued that CAC = 0 could be used as a “gatekeeper” to obviate the need to progress to more advanced anatomic or perfusion testing (e.g., by CTCA, PET, or coronary angiography). Similarly, Wang et al. reported outcomes in a group of patients with stable chest pain and non-calcified plaques (i.e., with zero CAC) and found them to have a favorable prognosis [23].

In their 1426-patient study (average age 50 years, 42% women), Patel et al. studied 5983 primary prevention patients at intermediate to high pretest risk undergoing PET/CT studies for suspected CAD [24]. CAC was zero in 22%, abnormal perfusion was noted in 19%, and a reduced myocardial blood flow reserve was found in 53%. CACS and myocardial blood flow reserve showed independent prognostic value for CV outcomes. The authors argued that microvascular dysfunction causing reduced myocardial blood flow reserve, also seen in 4 of 10 in the CAC = 0 setting, could lead to missing microvascular dysfunction and be associated with an increased risk of all-cause mortality.

Alternative mechanisms of myocardial infarction or ischemia are of particular interest in women, including SCAD, ANOCA, MINOCA, plaque erosion, and microvascular dysfunction, and these may not be predicted by CAC. Women comprise 87–95% of SCAD patients [7]. Moreover, women present more often with “open artery ischemia” syndromes, including angina (ANOCA) and myocardial infarction (MINOCA) [8,9,10]. Indeed, approximately two-thirds of MINOCA occur in women [8,10]. Microvascular dysfunction has been proposed as a mechanism of “open-artery ischemia”. Our MI event rates, although low, suggest that an important proportion of MIs in women are associated with non-obstructive CAD, as others also have reported [10,22].

Correlations with Present Study. The presentation of ischemic heart disease differs in several respects in women. They are more prone to angina with no obstructive coronary artery disease (ANOCA) and MINOCA [8,9,10]. They also are much more prone than men to spontaneous coronary artery dissection [7], which often develops in the setting of minimal or no CAD. Given these unique features in women, a value of CAC = 0 on prognosis requires further assessment.

We found, in this large cohort of women (19,495), 5400 with CAC = 0, that the medium-term prognosis with CAC = 0 is excellent and in line with reports from Mittal et al. and Blaha et al. [6,22]. Nevertheless, the potential for ANOCA, MINOCA, and spontaneous coronary artery dissection should be considered in individual patients.

The impact of CAC scoring in the elderly and of a zero CAC score requires additional investigative attention. The age in most published CAC study populations averaged in the 40s and 50s [13], so much less information has been obtained in those over 65, 75, and especially 80 years of age. In addition, a recent special communication suggested that “older adults may not see an expected benefit [of CAC testing] over [their] short time horizon or may already be taking lipid-lowering therapy, rendering a CAC score less valuable” [25]. With advancing age, coronary events increasingly compete with a growing number of other, non-CV-related events and non-coronary deaths, potentially rendering lipid-lowering therapy, initiated or withheld based on a CAC score, less impactful.

However, our observations indicate that the prognostic value of CAC is preserved in the elderly, with a very low incident rate of MI associated with CAC = 0. In support of our results, the MESA trial enrolled individuals up to 84 years of age and also noted CAC to be of prognostic value in the elderly [6].

Clinical Implications. The present study, which represents the largest single cohort of cardiac PET/CT patients, including a large number of women and older patients, confirms the prognostic value of CAC scoring in general and of a CAC score of zero in particular, regardless of sex or age. Thus, from the perspective of coronary events, CAC scoring is useful across the spectrum of middle-to-older ages and in women as well as in men when additional coronary risk refinement is desired. Its low cost (<USD 100 in our system) and low radiation exposure (<1 mSv) do not represent major barriers to its broader application where medically indicated.

Further, the finding that CAC is a prognosticator of total mortality (much of which is non-coronary related) is of recent interest [26] and may provide an additional indication for its application. Mechanisms for a general mortality benefit could be associations of CAC with disease in other vascular beds [27,28] and its proposed impact on immune surveillance for cancer risk [29,30]. Finally, if an outcomes study, now underway [31], shows equivalence (or superiority) of CAC-based versus risk-factor based guidance to initiate statin therapy, a recommendation for universal application in primary risk subjects may follow.

Strengths and limitations. This study’s strengths include our large and heterogeneous cohort population; this study’s age and sex stratification, allowing for focused clinical application; this study’s reinforcement of the utility of CAC = 0 in primary risk strategies, including implications for cholesterol lowering; and its total mortality comparisons between CAC categories. Its limitations include its retrospective, observational design, its limited follow-up period (≈2 years), and its limited information on concomitant therapy. These and its single-system cohort may limit generalizability to populations with differing race, ethnicity, and lifestyle distributions, and differing medical care systems. Also, we were able to extract less individual information from the much larger CAC > 0 population cohort, including cause-specific mortality and non-fatal coronary events. Mortality analysis by CAC > 0 severity also was not performed.

## 5. Conclusions

We asked whether sex or age impacts the prognostic value of a zero CAC score in patients at primary coronary risk. Based on a large experience in our integrated healthcare system, we found that a zero CAC score does predict an excellent prognosis for non-fatal MI or coronary death in both sexes and in older as well as in younger patients. CAC = 0 was more frequently observed in women despite an older average age. Rates of coronary events were at least as low in women as in men, with a trend to more MINOCA events and fewer obstructive coronary events in women. Further, a zero calcified plaque burden was found to predict a markedly lower risk of all-cause death in our overall population as well as in both sexes and in older and younger patients. Thus, CAC = 0 appears to represent a marker not only of coronary health but also of general vascular and non-cardiac organ health. Finally, these results raise the question of whether statin therapy, which is often recommended for the elderly by the age-dependent risk-factor equations (PCE, PREVENT), can be withheld in these CAC = 0 patients yet preserve favorable outcomes. This question is currently being tested in the CorCal Outcomes trial [31].

## Figures and Tables

**Table 1:** Baseline characteristics of CAC = 0 patients by sex.

Characteristic	Women with CAC = 0	Men with CAC = 0	*p*-Value
**Number of subjects, n (%)**	5400 (67.8)	2567 (32.2)	<0.001
**Age, mean (SD)**	60.5 (12.0)	53.8 (12.6)	<0.001
**Race, n (%)**			
**White/Caucasian**	4872 (90.2)	2234 (87.0)	<0.001
**African American (Black)**	48 (0.9)	67 (2.6)	<0.001
**Asian**	77 (1.4)	33 (1.3)	0.69
**American Indian/AK native**	64 (1.2)	16 (0.6)	0.03
**Multiple**	13 (0.2)	12 (0.5)	0.14
**Pacific Islander**	60 (1.1)	36 (1.4)	0.32
**Unknown**	266 (4.9)	169 (6.6)	0.003
**Family history of heart disease, n (%)**	2766 (55.2)	1052 (44.4)	<0.001
**Medical History, n (%)**			
**Hyperlipidemia**	3551 (65.8)	1551 (60.4)	<0.001
**Hypertension**	3653 (67.6)	1750 (68.2)	0.66
**Diabetes**	964 (18.5)	454 (18.5)	1.0
**Smoking history**	1335 (24.7)	964 (37.6)	<0.001
**Atrial fibrillation**	753 (13.9)	403 (15.7)	0.04
**COPD**	555 (10.3)	235 (9.2)	0.13
**Depression**	2510 (46.5)	720 (28.0)	<0.001
**Heart failure**	920 (17.0)	522 (20.3)	<0.001
**Renal failure**	1028 (19.0)	558 (21.7)	0.005
**Statins at discharge**	536 (9.9)	282 (11.0)	0.16
**Stroke**	664 (12.3)	228 (8.9)	<0.001
**Myocardial infarction**	259 (4.8)	160 (6.2)	0.01

Footnote: Results are given in numbers of subjects followed by percentages (in parentheses). Age is in average years with standard deviation. COPD = chronic obstructive pulmonary disease. *p*-values are for between-column comparisons.

**Table 2:** Baseline characteristics of CAC = 0 patients by age.

Characteristic	Patients Aged < 65 y old	Patients Aged ≥ 65 y old	*p*-Value
**Number of subjects, n (%)**	5185 (65.1)	2782 (34.9)	<0.001
**Age, median (IQR)**	52.0 (14)	70.0 (8)	<0.001
**Male, n (%)**	2014 (38.8)	553 (19.9)	<0.001
**Female, n (%)**	3171 (61.2)	2229 (80.1)	<0.001
**Race, n (%)**			
**White/Caucasian**	4507 (86.9)	2599 (93.4)	<0.001
**African American (Black)**	96 (1.9)	19 (0.7)	<0.001
**Asian**	75 (1.4)	35 (1.3)	0.56
**American Indian/AK native**	65 (1.3)	15 (0.5)	0.003
**Multiple**	16 (0.3)	9 (0.3)	1.0
**Pacific Islander**	83 (1.6)	13 (0.5)	<0.001
**Unknown**	343 (6.6)	92 (3.3)	<0.001
**Family history of heart disease**	2539 (53.3)	1279 (48.8)	0.01
**Medical history, n (%)**			
**Hyperlipidemia**	2999 (57.8)	2103 (75.6)	<0.001
**Hypertension**	3328 (64.2)	2075 (74.6)	<0.001
**Diabetes**	983 (19.9)	435 (16.1)	<0.001
**Smoking history**	1712 (33.0)	587 (21.1)	<0.001
**Atrial Fibrillation**	499 (9.6)	657 (23.6)	<0.001
**COPD**	433 (8.4)	357 (12.8)	<0.001
**Depression**	2137 (41.2)	1093 (39.3)	0.10
**Heart Failure**	853 (16.5)	589 (21.2)	<0.001
**Renal failure**	818 (15.8)	768 (27.6)	<0.001
**Statins at discharge**	546 (10.5)	272 (9.8)	0.31
**Stroke**	405 (7.8)	487 (17.5)	<0.001
**Myocardial infarction**	265 (5.1)	154 (5.5)	0.45

Footnote: Results are given in numbers of subjects followed by percentages (in parentheses). Age is in median years and interquartile ranges. COPD = chronic obstructive pulmonary disease.

## Data Availability

The data underlying this article cannot be shared publicly due to US privacy laws. The data are available upon reasonable request to the corresponding author.

## Supplementary Materials

The following supporting information can be downloaded at https://www.mdpi.com/article/10.3390/jcm14176260/s1: Supplementary Table S1. All-cause death binary logistic regression models, Supplementary Table S2. Baseline characteristics in CAC > 0 subjects by sex, Supplementary Table S3. Baseline characteristics in CAC > 0 subjects by age.

## Abbreviations

The following abbreviations are used in this manuscript:

CAC = 0	Coronary artery calcium score of zero
CAC	Coronary artery calcium
CAD	Coronary artery disease
PET/CT	Positron emission tomography/computed tomography
CV	Cardiovascular
MI	Myocardial infarction
eMR	Electronic medical records
MINOCA	MI with no obstructive CAD
ANOCA	Angina with no obstructive coronary artery disease
