# Supplementary material for: Does Sex or Age Impact the Prognostic Value of a Zero Coronary Artery Calcium Score?
Source: J Clin Med. 2025 Sep 4;14(17):6260. doi: 10.3390/jcm14176260 (PMC12429088; doi:10.3390/jcm14176260)
Supplement: Supplementary file 1 [file jcm-14-06260-s001.zip › jcm-3796652-supplementary.pdf]

Sex or Age Impact the Prognostic Value of a Zero Coronary Artery Calcium Score?

Jeffrey L Anderson [1,2*], Dave S Collingridge [1], Viet T Le [1,3], Leslie Iverson [1], Jospeh B Muhlestein [1,2], [1] Intermountain Heart Institute, Intermountain Health, Salt Lake City, UT;

Supplementary Materials.

Supplementary Table S1. All-Cause Death Binary Logistic Regression Models

All Patients			
**Variable**	**p-value**	**OR**	**95% CI for OR**
CAC (>0)	<0.001	2.31	2.00 – 2.66
Sex (male)	0.004	1.12	1.04 – 1.21
Age (≥65)	<0.001	1.93	1.76 – 2.10
Diabetes	<0.001	1.50	1.39 – 1.62
Hyperlipidemia	<0.001	0.76	0.69 – 0.84
Hypertension	<0.001	1.46	1.28 – 1.68
Smoking History	0.001	1.15	1.06 – 1.25
Renal Failure	<0.001	1.87	1.73 – 2.03
COPD	<0.001	1.61	1.47 – 1.76
Heart Failure	<0.001	2.03	1.88 – 2.19
Females			
**Variable**	**p-value**	**OR**	**95% CI for OR**
CAC (>0)	<0.001	2.34	1.96 – 2.80
Age (≥65)	<0.001	1.74	1.51 – 1.99
Diabetes	<0.001	1.59	1.41 – 1.79
Hyperlipidemia	<0.001	0.73	0.62 – 0.85

Hypertension	<0.001	1.57	1.27 – 1.93
Smoking History	0.12	1.10	0.98 – 1.25
Renal Failure	<0.001	1.83	1.62 – 2.05
COPD	<0.001	1.53	1.33 – 1.74
Heart Failure	<0.001	2.29	2.03 – 2.57
Males			
**Variable**	**p-value**	**OR**	**95% CI for OR**
CAC (>0)	<0.001	2.27	1.79 – 2.88
Age (≥65)	<0.001	2.05	1.83 – 2.31
Diabetes	<0.001	1.42	1.28 – 1.58
Hyperlipidemia	0.001	0.79	0.69 – 0.90
Hypertension	<0.001	1.40	1.17 – 1.67
Smoking History	0.002	1.18	1.07 – 1.31
Renal Failure	<0.001	1.91	1.72 – 2.12
COPD	<0.001	1.67	1.48 – 1.89
Heart Failure	<0.001	1.84	1.66 – 2.05
<65 Years of Age			
**Variable**	**p-value**	**OR**	**95% CI for OR**
CAC (>0)	<0.001	2.18	1.76 – 2.70
Sex (male)	0.67	0.97	0.83 – 1.13
Diabetes	<0.001	1.57	1.33 – 1.84
Hyperlipidemia	0.26	0.90	0.75 – 1.08
Hypertension	0.06	1.27	0.99 – 1.64
Smoking History	<0.001	1.39	1.19 – 1.63

Renal Failure	<0.001	3.34	2.84 – 3.93
COPD	<0.001	1.55	1.28 – 1.89
Heart Failure	<0.001	1.92	1.63 – 2.26
≥65 Years of Age			
**Variable**	**p-value**	**OR**	**95% CI for OR**
CAC (>0)	<0.001	2.35	1.94 – 2.85
Sex (male)	<0.001	1.18	1.08 – 1.28
Diabetes	<0.001	1.45	1.32 – 1.59
Hyperlipidemia	<0.001	0.70	0.62 – 0.80
Hypertension	<0.001	1.51	1.29 – 1.78
Smoking History	0.19	1.07	0.97 – 1.17
Renal Failure	<0.001	1.57	1.43 – 1.71
COPD	<0.001	1.64	1.48 – 1.81
Heart Failure	<0.001	2.04	1.86 – 2.23

Supplementary Table S2. Baseline Characteristics in CAC>0 Subjects by Sex

Characteristic	Women with CAC > 0	Men with CAC > 0	P-value
Number of subjects, n (%)	14,095 (44.0)	17,956 (56.0)	<0.001
Age, mean (SD)	69.4 (10.5)	66.3 (11.2)	<0.001
Race, n(%)			
-White/Caucasian	12,863 (91.3)	16,255 (90.5)	0.03
-African American (Black)	96 (0.7)	181 (1.0)	0.002
-Asian	181 (1.3)	231 (1.3)	1.0
-American Indian/AK native	102 (0.7)	115 (0.6)	0.41
-Multiple	34 (0.2)	33 (0.2)	0.32
-Pacific Islander	276 (2.0)	346 (1.9)	0.87
-Unknown	543 (3.9)	795 (4.4)	0.01
Family history of heart disease, n(%)	7663 (57.8)	7788 (46.3)	<0.001
Medical History, n (%)			
-Hyperlipidemia	11,771 (83.5)	14,473 (80.6)	<0.001
-Hypertension	12,077 (85.7)	14,993 (83.5)	<0.001
-Diabetes	4046 (29.5)	5232 (30.1)	0.26
-Smoking history	4175 (29.6)	6821 (38.0)	<0.001
-Atrial fibrillation	3176 (22.5)	4436 (24.7)	<0.001
-COPD	2556 (18.1)	2723 (15.2)	<0.001
-Depression	6377 (45.2)	4686 (26.1)	<0.001
-Heart failure	3816 (27.1)	4711 (26.2)	0.10
-Renal failure	4532 (32.2)	5549 (30.9)	0.02
-Statins at discharge	2363 (16.8)	3207 (17.9)	0.01
-Stroke	2913 (20.7)	2896 (16.1)	<0.001
-Myocardial infarction	997 (7.1)	1294 (7.2)	0.66

Supplementary Table S3. Baseline Characteristics in CAC>0 Subjects by Age

Characteristic	Patients Aged <65y old	Patients Aged ≥65y old	P-value
Number of subjects, n (%)	11,248 (35.1)	20,803 (64.9)	<0.001
Age, median (IQR)	58.0 (9)	73.0 (9)	<0.001
Male , n (%)	7222 (64.2)	10,734 (51.6)	<0.001
Female, n (%)	4026 (35.8)	10,069 (48.4)	<0.001
Race, n (%)			
-White/Caucasian	9657 (85.9)	19,461 (93.5)	<0.001
-African American (Black)	185 (1.6)	92 (0.4)	<0.001
-Asian	182 (1.6)	230 (1.1)	<0.001
-American Indian/AK native	123 (1.1)	94 (0.5)	<0.001
-Multiple	29 (0.3)	38 (0.2)	0.20
-Pacific Islander	441 (3.9)	181 (0.9)	<0.001
-Unknown	631 (5.6)	707 (3.4)	<0.001
Family history of heart disease	5796 (55.7)	9655 (49.1)	<0.001
Medical history, n (%)			
-Hyperlipidemia	8334 (74.1)	17,910 (86.1)	<0.001
-Hypertension	9041 (80.4)	18,029 (86.7)	<0.001
-Diabetes	3660 (34.0)	5618 (27.6)	<0.001
-Smoking history	4295 (38.2)	6701 (32.2)	<0.001
-Atrial Fibrillation	1554 (13.8)	6058 (29.1)	<0.001
-COPD	1411 (12.5)	3868 (18.6)	<0.001
-Depression	4056 (36.1)	7007 (33.7)	<0.001
-Heart Failure	2527 (22.5)	6000 (28.8)	<0.001
-Renal failure	2782 (24.7)	7299 (35.1)	<0.001
-Statins at discharge	1952 (17.4)	3618 (17.4)	0.95
-Stroke	1203 (10.7)	4606 (22.1)	<0.001
-Myocardial infarction	757 (6.7)	1534 (7.4)	0.04
